# Associations between EBV and CMV Seropositivity, Early Exposures, and Gut Microbiota in a Prospective Birth Cohort: A 10-Year Follow-up

**DOI:** 10.3389/fped.2016.00093

**Published:** 2016-08-31

**Authors:** Claudia Carvalho-Queiroz, Maria A. Johansson, Jan-Olov Persson, Evelina Jörtsö, Torbjörn Kjerstadius, Caroline Nilsson, Shanie Saghafian-Hedengren, Eva Sverremark-Ekström

**Affiliations:** ^1^Department of Molecular Biosciences, Wenner-Gren Institute, Stockholm University, Stockholm, Sweden; ^2^Department of Mathematics, Stockholm University, Stockholm, Sweden; ^3^Department of Clinical Science and Education, Karolinska Institute, Stockholm, Sweden; ^4^Sachs’ Children’s and Youth Hospital, Stockholm South General Hospital, Stockholm, Sweden; ^5^Department of Clinical Virology and Microbiology, Karolinska University Laboratory, Solna, Sweden; ^6^Department of Clinical Microbiology, Central Hospital, Karlstad, Sweden; ^7^Department of Women’s and Children’s Health, Paediatric Oncology Unit, Astrid Lindgren Children’s Hospital, Karolinska Institute, Sweden

**Keywords:** healthy children, EBV, CMV, seroprevalence, risk factors, gut microbiota, *S. aureus*, *Lactobacillus*

## Abstract

Early-life infections with persistent Epstein–Barr virus (EBV) and cytomegalovirus (CMV) are delayed in affluent countries, probably due to alterations in early environmental exposures, such as maternal age, siblings, and day-care attendance. We have previously reported that the timing of EBV and CMV contraction is related both to allergic sensitization and changes in functional competence of immune cells, while the presence/absence of lactobacilli [*Lactobacillus (L.) casei, L. paracasei*, and *L. rhamnosus*] or *Staphylococcus (S.) aureus* in feces is related to the risk for allergy. Here, we used the same prospective longitudinal birth cohort of children to investigate early-life environmental exposures and their influence on EBV and CMV contraction over time. Since gut microbes also belong to this category of early exposures, we investigated their association with herpesvirus contraction. Our results show that these two viruses are acquired with different kinetics and that EBV and CMV seroprevalence at 10 years of age was 47 and 57%, respectively. We also observed that a delayed EBV or CMV infection was associated with older maternal age [time ratio (TR) 1.14, 95% confidence interval (CI) 1.07–1.21, *P*_adj_ < 0.001 and TR 1.09, CI 1.03–1.16, *P*_adj_ = 0.008, respectively]. Further, we present the novel finding that *S. aureus* colonization reduced the time to CMV acquisition (TR 0.21, CI 0.06–0.78, *P*_adj_ = 0.02). Together, these findings suggest that there is a relationship between timing of herpesvirus acquisition and early-life immune modulating exposures, which interestingly also includes the early infant gut microbiota.

## Introduction

Epstein–Barr virus (EBV) and cytomegalovirus (CMV) are herpesviruses commonly contracted during infancy, and they are prevalent in the human population worldwide. They belong to what is referred to as the human virome or virobiota – a collection of both eukaryotic and prokaryotic viruses are found in humans (1–3). The majority of adults (over 90%) are considered to be seropositive for EBV (4, 5), and the transmission commonly occurs *via* saliva (6). In early childhood, primary infection with EBV is commonly mild or even asymptomatic, while during adolescence or in adults, the EBV infection more often manifests as acute infectious mononucleosis (5, 7). In relation to CMV, between 60 and 90% of adults are considered to be seropositive, however not without substantial geographic variation that is associated with socioeconomic status (8). CMV transmission occurs through various body fluids including maternal genital secretions at birth and breast-milk, as well as *via* urine from children and adults (9, 10). EBV can infect B-lymphocytes and epithelial cells (11), while myeloid cells are the main CMV reservoirs (12). After primary infection, EBV and CMV establish latency and persist for life. Both viruses are then chronically or intermittently shed for the lifetime of the host, and their re-activation is under tight immunological control. Still, herpesviruses can be a serious threat to the immunocompromised host (2, 4).

There has been a marked change in life-style and environmental exposures in many parts of the world during the last decades, which has been accompanied by a steep increase of immune-mediated disorders, such as allergy and asthma. Also, the higher sanitation standards of westernized countries and prompt antibiotic availability are believed to cause alterations in our exposure to beneficial microbes, which may be disadvantageous to immune development and maturation (2, 13–15). Using a well-characterized prospective longitudinal birth cohort, we have previously reported that EBV and CMV seropositivity relates both to allergic sensitization (16, 17) and modulated immune function in children (18–20). The presence of early-life microbiota was also investigated in this cohort, where a group of lactobacilli [*Lactobacillus (L.) casei, L. paracasei*, and *L. rhamnosus*] and *Staphylococcus (S.) aureus* DNA were related to opposite allergic profiles at 5 years of age (21). As the order and timing of colonization with these microbial agents has been suggested to affect functional capacity of the infant immune system (15–21), herein we investigated the seropositivity for EBV and CMV at four time points up to 10 years of age in the same children and assessed the possible association between environmental exposures, including gut microbiota colonization following birth and the acquisition of herpesviruses over a 10-year period.

## Materials and Methods

### Ethical Statement

The study was approved by the Human Ethics Committee at Huddinge University Hospital, Stockholm (Dnr 75/97, 331/02, 2007/858-31/2), and the parents provided their informed verbal consent. No written documentation of the participants informed approval was required, which was agreed to by the Human Ethics Committee and was according to the regulations at the time of the initiation of the study.

### Subjects

Our study included subjects from a prospective birth cohort of 281 children born between 1997 and 2000 in the Stockholm area (16, 17, 21). The demographic information from the study population is presented in Table 1. Two thirds of the children had parental allergic heredity (either from the mother or from both parents), while the remaining third had no allergic heredity. The parents were asked at the maternity wards to participate with their child in the study and were given the possibility of calling an experienced pediatric nurse with questions about their child at any time. Data collection on exposures (breastfeeding, day care, etc.) was described in detail previously (16, 17, 21).

**Table 1:** **Demographics of study population**.

Characteristics	Total
**Children, *n***	281
**Maternal age at delivery**	
Years, median (min–max)	31 (21–44)
**Child gender**^a^	
Boys, *n* (%)	140 (49.8)
Girls, *n* (%)	139 (49.2)
**Delivery mode**	
Vaginal, *n* (%)	238 (84.7)
C-section, *n* (%)	43 (15.3)
**Exclusive breastfeeding**^b^	
Months, median (range)	4 (0–10)
**Day-care start**^c^	
Months, median (min–max)	18 (12–26)
**Siblings**^d^	
Number, median (min–max)	0 (0–5)

*Data missing from ^a^n = 2, ^b^n = 1, ^c^n = 17, and ^d^n = 35 individuals*.

### Serological Methods for Determinations of EBV and CMV Infections

Venous blood samples were drawn at several time points in order to evaluate the presence of IgG antibodies against EBV and CMV. For the presence of IgG antibodies against both EBV and CMV at 1, 2, and 5 years, the protocol was described previously (16, 17). To determine the herpesvirus serostatus at 10 years of age, IgG against EBV viral-capsid antigen as well as EBV nuclear antigen-1 was analyzed *via* immunofluorescence assays (22), and IgG against CMV was determined with an in-house CMV-IgG ELISA according to a protocol described elsewhere (23). Serum samples were analyzed in dilutions of 1/50, 1/100, and 1/1000. An absorbance value of 0.20 was taken as a positive reaction. Positivity in dilution 1/50, but not in 1/100, was considered as gray zone.

### Lactobacilli and *Staphylococcus (S.) aureus* Detection in Fecal Samples

The presence and the relative frequencies (expressed as %) of DNA of commensals were detected previously by us (21, 24) through real-time PCR in fecal samples of the children in the cohort at 1 and 2 weeks and at 1 and 2 months of age, providing that they were vaginally delivered, fully breastfed for at least 3 months, received no antibiotics treatment, and had fecal samples available for testing. Different species of *Bifidobacterium, S. aureus*, a group of lactobacilli [*Lactobacillus (L.) casei, L. paracasei*, and *L. rhamnosus*], and *Clostridium difficile* were evaluated on those studies (21, 24). In this present study, however, we focused our analyses on the lactobacilli group and *S. aureus* only, due to their notable opposite associations with the immune profile and allergy prevalence during infancy and childhood (21, 24–27).

### Statistics

Association analyses between infection (EBV/CMV) and explanatory variables were performed separately for each age group since some children did not have complete data from all 4 years. Associations were estimated as odds ratios (ORs) using univariate logistic regression models, with EBV (0/1) or CMV (0/1) as outcome. In an effort to use the information from all four time points in one analysis, an interval censored survival analysis was also performed. Here, the input data for each child were the time interval when the infection occurred [e.g., a child not infected at year 2 and infected at year 4 get a time interval of (2, 4), and a child uninfected during the four time points get a time interval of >10]. Such time intervals were, despite missing data, possible to decide for the majority of children. A two-parameter gamma distribution, parameterized as an accelerated failure time model, was used to model the infection times. The effect of an explanatory variable on the infection time is manifested as a time ratio (TR) and shows how an explanatory variable speeds up or delays the expected infection time. ORs and TRs were reported with a corresponding 95% confidence interval (CI), and a *P*-value for a test of the hypothesis that the OR/TR is equal to 1. Due to the explorative nature of testing associations between EBV and CMV infections and early-life exposures (Table 3), results were adjusted for multiple analyses according to the Bonferroni correction, whereby *P* < 0.01 was considered as statistically significant. In the remaining analyses, statistical significance was declared when *P* ≤ 0.05. The statistical computations were performed with Stata 13 (StataCorp LP, USA).

## Results

### EBV and CMV Seroprevalence during the First 10 Years of Life

**Figure 1:**
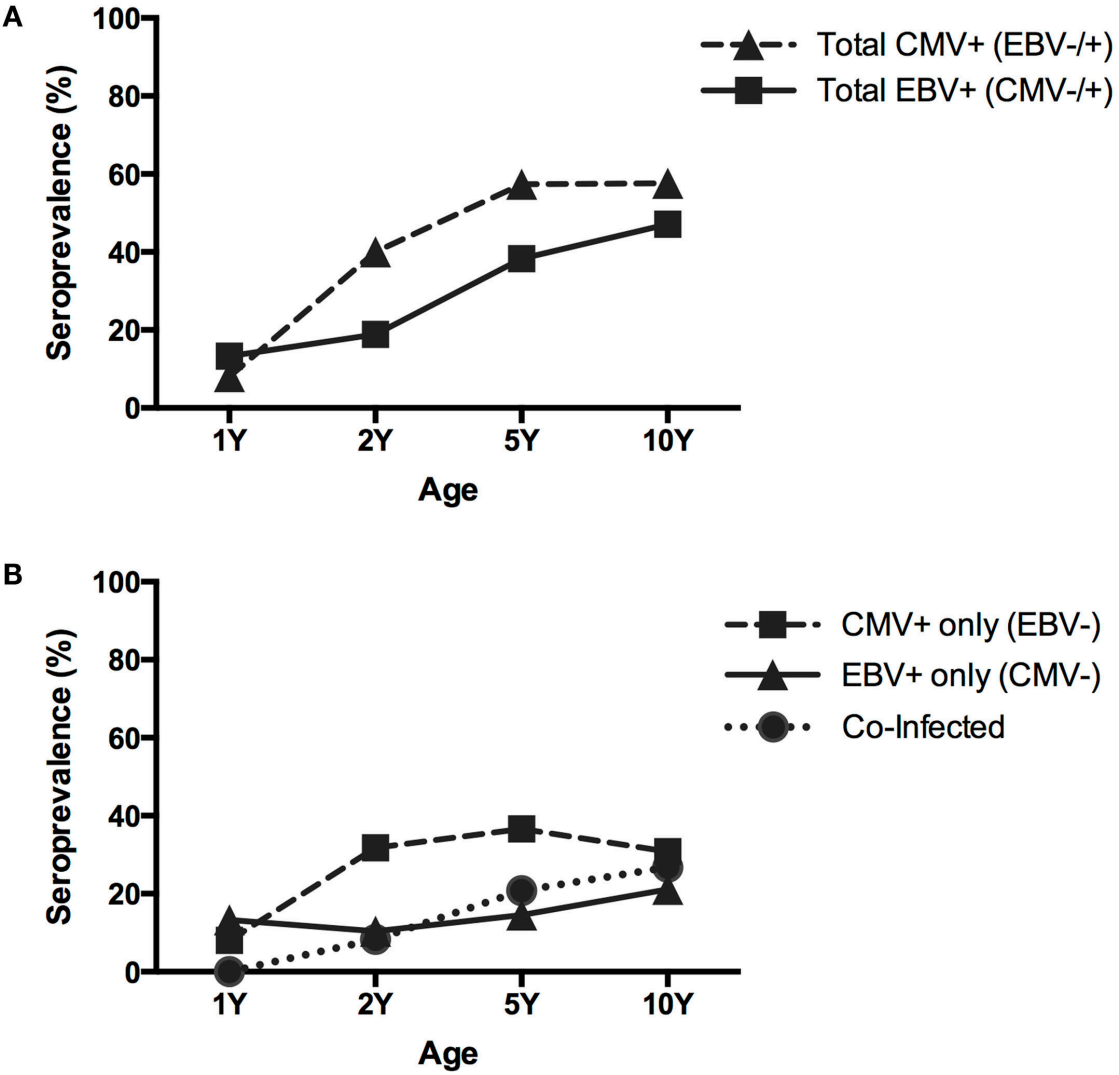
**Seropositivity of CMV and EBV**. **(A)** Total CMV and total EBV seropositivity. **(B)** Single CMV seropositivity, single EBV seropositivity, and co-infection with EBV and CMV. Y: years.

The seroprevalence of EBV gradually increased up to 10 years of age from 13% at 1 year, 18.9% at 2 years, 38.3% at 5 years, and up to 47.1% at 10 years of age. In contrast, for CMV, a rapid increase between 1 and 2 and a clear plateau between 5 and 10 years of age was observed, where 8% of the children were positive at 1 year, 40.0% at 2 years, 57.4% at 5 years, and 57.6% at 10 years of age (Table 2; Figure 1A). Upon subdividing the children into three groups based on the presence of EBV or CMV alone or co-infection with both viruses, we observed that 8.2% were positive for both viruses at 2 years, 20.9% at 5 years, and 26.9% at 10 years of age. None of the children at 1 year were co-infected with CMV and EBV (Figure 1B).

**Table 2:** **Herpesvirus seroprevalence during the first 10 years of life in children born in Stockholm**.

	Age			
Children	1 year^a^	2 years^b^	5 years^c^	10 years^d^
EBV seropositive (%)	13.0	18.9	38.3	47.1
CMV seropositive (%)	7.8	40.0	57.4	57.6

*A total of *n* = 281 individuals were followed from birth to 10 years of age. Plasma was available for determination of EBV and CMV seropositivity from ^a^*n* = 115, ^b^*n* = 241, ^c^*n* = 215, and ^d^*n* = 207 children. Children with inconclusive samples for either EBV (*n* = 3) or CMV (*n* = 9) were excluded from all time points*.

### EBV and CMV Acquisition Is Associated with Maternal Age

In order to establish if there were any associations between EBV or CMV acquisition and early-life environmental exposures, the time to infection with these viruses was investigated as the outcome (Table 3). We found a statistically significant increase in time to EBV infection (i.e., delayed infection) per year increase of the maternal age (TR 1.14, CI 1.07–1.21, *P* < 0.001), which was sustained following adjustment for all included variables (Table 3). Following exclusion of data from 1-year-old time point, the following adjusted *P*-values were generated for EBV: <0.001 (maternal age), 0.73 (delivery mode), 0.20 (day-care start age), 0.32 (exclusive breastfeeding), and 0.92 (older siblings, data not shown). When the time to infection with CMV was investigated as the outcome (Table 3), a delay in time to CMV infection per year increase of the mother’s age (TR 1.09, CI 1.03–1.16, *P* = 0.004) was observed, which was statistically significant after adjustment for all included variables. Following exclusion of data from 1-year-old time point, the following adjusted *P*-values were generated for CMV: <0.008 (maternal age), 0.59 (delivery mode), 0.90 (day-care start age), 0.91 (exclusive breastfeeding), and 0.09 (older siblings, data not shown).

**Table 3:** **Associations between early-life exposures and EBV and CMV seropositivity**.^a^

		EBV				CMV		
Variable^b^	*N*	TR (95% CI)	*P*	*P*_adj_	*N*	TR (95% CI)	*P*	*P*_adj_
Maternal age	265	1.14 (1.07–1.21)	**<0.001**	**<0.001**	265	1.09 (1.03–1.16)	**0.004**	**0.008**
Delivery mode	265	0.89 (0.45–1.75)	0.74	0.79	265	0.82 (0.41–1.64)	0.57	0.51
Day-care start age	228	1.09 (1.00–1.18)	0.05	0.20	228	1.03 (0.94–1.13)	0.55	0.80
Exclusive breastfeeding	265	1.02 (0.87–1.19)	0.82	0.43	265	0.97 (0.83–1.13)	0.67	0.86
Older siblings	246	1.13 (0.81–1.56)	0.48	0.97	246	1.56 (1.07–2.26)	0.02	0.06

*^a^The effect of an explanatory variable on the infection time manifested as a time ratio (TR); *N*: number of individuals with data available for each given variable; CI: confidence interval range; *P*_adj_: *P*-values adjusted for all^b^ included variables. Bolded *P*-values: statistically significant following Bonferroni correction*.

When evaluating associations for each age separately, the odds of EBV infection at 2, 5, and 10 years of age decreased with increased maternal age at time of birth, i.e., EBV infection was delayed in children with older mothers (Table S1 in Supplementary Material). When evaluating the associations between maternal age and likelihood of CMV infection for each age separately, however, no statistically significant associations were found (Table S2 in Supplementary Material). The odds of being CMV infected at 2 years of age was, however, low when the number of older siblings increased (Table S2 in Supplementary Material). We did not observe any notable differences in socioeconomic status between the group of children that were infected with herpesviruses early (i.e., at age 2 years) and those who remained seronegative at the age of 10 years (Figure S1 in Supplementary Material).

### Timing of CMV Acquisition Is Associated with Early-Life Gut Microbes

Studies in mice reveal that the gut microbiota can influence the susceptibility to viral infections (28, 29), but whether this is true also in humans is not known. We investigated the associations between EBV and CMV and the presence of two different types of bacteria commonly present in the neonatal gut but with opposite effects on immune function – a group of lactobacilli (*L. casei, L. paracasei*, and *L. rhamnosus*) and *S. aureus* (25, 27). There were no statistically significant associations between early colonization with lactobacilli and EBV or CMV infections when investigating time of infection as the outcome (Table 4). Interestingly, *S. aureus* colonization did not seem to influence the time to EBV infection, while it was significantly associated with earlier CMV infection if children were colonized with *S. aureus* on two or more occasions (Table 4, TR 0.21, CI 0.06–0.78, *P* = 0.02), which sustained following adjustment for maternal age (*P*_adj_ = 0.02, Table 4). Following exclusion of data from 1-year-old time point, *S. aureus* colonization was still associated with earlier CMV infection if children were colonized with *S. aureus* on two or more occasions (*n* = 65, TR 0.12, CI 0.01–1.01, *P*_adj_ = 0.05).

**Table 4:** **Association between gut microbes and time to EBV and CMV acquisition**.^a^

		EBV				CMV		
Detection after birth	*N*	TR (95% CI)	*P*	*P*_adj_	*N*	TR (95% CI)	*P*	*P*_adj_
**Lactobacilli**								
1 week, presence	68	1.15 (0.29–4.49)	0.84	0.98	68	3.1 (0.77–12.47)	0.11	0.10
2 weeks, presence	66	0.62 (0.18–2.12)	0.44	0.24	66	1.45 (0.49–4.27)	0.50	0.46
1 month, presence	63	0.68 (0.21–2.14)	0.50	0.32	63	2.00 (0.61–6.58)	0.25	0.25
2 months, presence	65	2.01 (0.55–7.31)	0.29	0.11	65	1.73 (0.57–5.28)	0.33	0.33
Occasions, 2 or more	67	1.56 (0.44–5.49)	0.49	0.76	67	1.83 (0.60–5.58)	0.29	0.27
***S. aureus***								
1 week, presence	68	1.55 (0.46–5.20)	0.48	0.42	68	0.39 (0.12, 1.25)	0.11	0.11
2 weeks, presence	66	0.44 (0.11–1.81)	0.25	0.29	66	0.21 (0.06, 0.78)	**0.02**	**0.02**
1 month, presence	63	0.97 (0.26–3.56)	0.96	0.69	63	0.36 (0.09, 1.47)	0.15	0.16
2 months, presence	65	0.87 (0.23–3.29)	0.83	0.77	65	0.57 (0.16, 1.98)	0.37	0.38
Occasions, 2 or more	67	1.02 (0.28–3.70)	0.97	0.50	67	0.35 (0.10, 1.21)	0.10	0.10

*^a^All subjects were vaginally delivered, fully breastfed for a minimum of 3 months, and did not undergo any antibiotics treatment at the time of fecal sampling. The effect of an explanatory variable on the infection time manifested as a time ratio (TR); *N*: number of individuals with data available for each given variable; CI: confidence interval range; *P*_adj_: *P*-values adjusted for mother’s age. Bolded *P*-values: statistically significant*.

No statistically significant differences with regard to maternal age, day-care starting age, or number of siblings among the groups assessed for colonization with the Lactobacilli or *S. aureus* were found (data not shown). Further, on analyzing association between early gut colonization and herpesvirus infection in relation to each age group separately (Tables S3 and S4 in Supplementary Material), *S. aureus* colonization was related to increased odds of CMV seropositivity at 5 and 10 years of age (OR 3.16, CI 1.00–10.00, *P*_adj_ < 0.05 and OR 4.11, CI 1.20–14.13, *P*_adj_ = 0.02, respectively; Table S4 in Supplementary Material). Following exclusion of data from 1-year-old time point, *S. aureus* colonization was still associated with increased odds of CMV seropositivity at 5 and 10 years of age (*n* = 56, OR 3.11, CI 0.98–9.87, *P*_adj_ = 0.05 and *n* = 55, OR 4.31, CI 1.22–15.18, *P*_adj_ = 0.02, respectively).

## Discussion

In our study, we took advantage of a longitudinal prospective birth cohort of children followed until 10 years of age, thereby providing a unique opportunity to investigate early environmental exposures and their possible influence on EBV and CMV infections over time. Our results show that these two viruses are acquired with different kinetics and that the EBV and CMV seroprevalence at age of 10 years was 47 and 57%, respectively. When relating these findings to maternal age as well as to early exposures such as duration of breastfeeding, siblings, and day-care attendance, we observed that a delayed EBV and CMV infection was associated with older maternal age. Further, we found associations between the early-life gut colonization and modulation of odds to contract CMV infection as *S. aureus* colonization early in life reduced the time to CMV acquisition.

Herpesvirus infections are delayed in affluent compared to developing countries (30). In line with these observations, we found relatively low herpesvirus seroprevalence in our study cohort of Swedish children, with around 57% CMV and 47.1% EBV seropositive children at 10 years of age. The prevalence of congenital CMV, which is observed when the virus is transmitted to the fetus upon primary infection, reactivation, or re-infection, has been shown to occur in 0.2–2% of pregnant women in westernized countries [reviewed in Ref. (31)], arguing against a major influence on our results. Also, to avoid misinterpretation of the seropositivity and/or infection kinetics due to interference with maternal herpesvirus-specific antibodies in the circulation of newborn children, we choose to exclude data concerning seropositivity at 6 months of age. In addition, similar *P*-values we generated when analyses were performed with or without EBV and CMV serostatus data from 1-year olds. When comparing the kinetics between EBV and CMV infections, our results showed that CMV was often acquired earlier in life than EBV, and CMV was also more common in our study population at 10 years of age than EBV. In agreement with our observations of the kinetics of the time of contracting EBV and CMV, which was most rapid during the first 2 years of life for both viruses, children are more likely to be exposed to these herpesviruses through their contact with family and day-care members early in life (9). The reason for CMV being the most prevalent of these two viruses at 2–10 years of age is not clear but may be in part connected to the maturation and expansion of virus-specific memory cells. In agreement with this, we have shown that CMV seropositive infants who are EBV naive have a large population of IFN-γ-producing T cells (19, 20), the presence of which associates with the inhibition of B-cell transformation upon *in vitro* EBV infection of B cells derived from children in this cohort (20). However, other factors such as differences in the prevalence of herpesviruses among the parents, transmission kinetics, the infectivity, and/or the reactivation potential of each virus may also play a role.

We also found that a delay in both EBV and CMV infections was related to older maternal age at the time of birth. Such associations with maternal age have been previously described for CMV, where the infection was more frequent in children whose mothers were 25 years or younger – which was suggested to be linked to higher CMV viral shedding in younger mothers (32). Alternatively, we can speculate that social and behavioral changes and/or altered hygiene practices with increased maternal age may also play a role in delayed infection pattern with herpesviruses. Whether other coupling factors other than those the results were adjusted for could explain this finding may be possible. Yet, socioeconomic inequality, which is a central risk factor for microbial infections, does not explain our findings since children that became seropositive for either herpesviruses at 2 years of age had similar socioeconomic status to those remaining seronegative at 10 years of age.

The microbiota plays an integral role in supplying important signals for immune priming, immune development, and affecting the susceptibility to pathogens (33, 34). The interactions between our commensal microbiota and bacterial pathogens, especially in early life, have been extensively pursued during the past decades (14, 35, 36), while the interplay between viruses and commensals has only recently begun to be appreciated (2, 3, 33, 37). Indeed, the influence of microbiota on viral infections can have a dual role: it can culminate in protection of the host against the virus and in promotion of the viral establishment (34, 37, 38). In our study, we investigated the presence of lactobacilli or *S. aureus* DNA in fecal samples as proxies for early-life microbiota, since we have earlier found in our cohort that these are linked to immunomodulatory properties during early childhood (21, 25). Our results indicate that an early *S. aureus* colonization decreased the time to CMV acquisition. This finding is intriguing in relation to earlier observations on increased risk of allergy in infants who were CMV infected but EBV naive (16). How microbes in the gut condition the host resistance against herpesviruses in humans remains to be addressed. Bleotu et al. found that infection of HeLa cells with herpes simplex virus (HSV) in the presence of inactivated *S. aureus* induced TNF, IL-6, and IL-8 cytokines, and suggested that bacterial antigens could contribute to altered herpesvirus cell binding and multiplication rate *in vitro* (39).

Some limitations should be acknowledged. As different species and/or strains of commensals can play a different role, a full microbiota analysis could bring more information about their composition and possible association with CMV, EBV, and other viruses, especially in a larger cohort of children. Also, it could be argued that the statistical model used here may lose stability due to the size of data sets. Nonetheless, using TR as our read-out enabled us to include additional subjects with occasional missing data. Further, interactions with viruses and bacteria could shape immune responses and have previously been described for better (40) or for worse (41, 42) but mainly in animal models. Therefore, despite the limited number of children and commensal species investigated here, our study is unique with its longitudinal approach with data sets following birth to 10 years of age and is among the first to describe an association between early infant gut colonization and timing of CMV contraction. Mechanisms behind the contribution of cross talk between herpesviruses and the gut microbiota for the development of an allergic phenotype remain yet unclear and should be elucidated in future studies.

In conclusion, we report that both EBV and CMV seropositivity occurs relatively late in Swedish children and that maternal age seems to be an important factor for the odds of acquiring EBV or CMV infection. Further, our data suggest that a connection between harboring certain gut species and tuning of host reactivity against herpesviruses may exist, which could ultimately influence maturation and functional competence of the immune system and potentially also allergic sensitization during childhood.

## Author Contributions

CC-Q performed data collection and experimental analyses and wrote the manuscript. MJ performed data collection and experimental analyses. J-OP performed statistical analyses. EJ performed experimental analyses. TK performed experimental analyses and contributed reagents/materials/analysis tools. CN conceptualized and designed the study and contributed reagents/materials/analysis tools. SS-H conceptualized and designed the study, performed experimental analyses, and wrote the manuscript. ES-E conceptualized and designed the study, contributed reagents/materials/analysis tools, performed experimental analyses, and wrote the manuscript.

## Conflict of Interest Statement

The authors declare that the research was conducted in the absence of any commercial or financial relationships that could be construed as a potential conflict of interest. The reviewer PK and handling Editor declared their shared affiliation, and the handling Editor states that the process nevertheless met the standards of a fair and objective review.
