# Supplementary material for: Associations between EBV and CMV Seropositivity, Early Exposures, and Gut Microbiota in a Prospective Birth Cohort: A 10-Year Follow-up
Source: Front Pediatr. 2016 Aug 31;4:93. doi: 10.3389/fped.2016.00093 (PMC5006634; doi:10.3389/fped.2016.00093)
Supplement: Supplementary file 1 [file Table_1.DOCX]

Supplementary Material

Associations between EBV and CMV seropositivity, early exposures and gut microbiota in a prospective birth cohort: a 10 year follow-up

Claudia Carvalho-Queiroz^1^, Maria A. Johansson^1#^, Jan-Olov Persson^2#^, Evelina Jörtsö^3, 4^, Torbjörn Kjerstadius^5, 6^, Caroline Nilsson^3, 4^, Shanie Saghafian-Hedengren^7§^ and Eva Sverremark-Ekström^1§*^

*** Correspondence:** Eva Sverremark-Ekström, Stockholm University, Department of Molecular Bioscience, The Wenner-Gren Institute, Svante Arrhenius väg 20 C, 106 91 Stockholm, Sweden, Telephone: +46 8 16 41 78, Fax: +46 8 612 95 42, E-mail: eva.sverremark@ su.se

# Supplementary Table S1

| **Table S1: Relationship Between Children’s Early-Life Exposures and EBV Serostatus*** | | | | | | | | | | |
| --- | --- | --- | --- | --- | --- | --- | --- | --- | --- | --- |
|  |  | | 1Y |  |  |  | 2Y |  |  |  |
| Variable# | | *N* | OR (95% CI) | *P* | *P_adj_* | *N* | OR (95% CI) | *P* | *P_adj_* |  |
| **Maternal age** | | 115 | 1.01 (0.89-1.13) | .91 | .56 | 241 | 0.83 (0.77-0.91) | **<.001** | **<.001** |  |
| **Delivery mode** | | 100 | 1 (1-1) | 1 | 1.0 | 241 | 0.71 (0.28-1.82) | .48 | .66 |  |
| **Day-care start** | | 94 | 0.97 (0.79-1.21) | .82 | .87 | 210 | 0.95 (0.85-1.07) | .42 | .97 |  |
| **Exclusive breastfeeding** | | 115 | 0.76 (0.56-1.03) | .08 | .17 | 241 | 1.11 (0.91-1.36) | .31 | .17 |  |
| **Older Siblings** | | 107 | 1.15 (0.61-2.16) | .67 | .90 | 241 | 0.85 (0.56-1.28) | .43 | .48 |  |
|  | |  |  |  |  |  |  |  |  |  |
|  | |  | 5Y |  |  |  | 10Y |  |  |  |
| Variable# | | *N* | OR (95% CI) | *P* | *P_adj_* | *N* | OR (95% CI) | *P* | *P_adj_* |  |
| **Maternal age** | | 215 | 0.88 (0.82-0.94) | **<.001** | **<.001** | 207 | 0.89 (0.83-0.96) | **<.001** | **.009** |  |
| **Delivery mode** | | 215 | 1.34 (0.65-2.76) | .43 | .24 | 207 | 1.00 (0.45-2.23) | 1 | .77 |  |
| **Day-care start** | | 187 | 0.95 (0.86-1.04) | .28 | .47 | 181 | 0.91 (0.82-1.00) | .05 | .28 |  |
| **Exclusive breastfeeding** | | 215 | 1.09 (0.91-1.30) | .36 | .06 | 207 | 1.00 (0.84-1.18) | .99 | .66 |  |
| **Older Siblings** | | 202 | 0.78 (0.53-1.14) | .19 | .67 | 194 | 0.96 (0.68-1.35) | .80 | .42 |  |
| *Univariate analysis of association, for each age separately. Y: Years of age; N: Number of observations; OR: Odds ratio;  CI: Confidence interval range; *P_adj_*: values adjusted for included variables^#^. Bolded *P*-values: statistically significant if *P*<0.01, following Bonferroni correction. . | | | | | | | | | |  |
