# Supplementary material for: Associations between EBV and CMV Seropositivity, Early Exposures, and Gut Microbiota in a Prospective Birth Cohort: A 10-Year Follow-up
Source: Front Pediatr. 2016 Aug 31;4:93. doi: 10.3389/fped.2016.00093 (PMC5006634; doi:10.3389/fped.2016.00093)
Supplement: Supplementary file 2 [file Table_2.DOCX]

Supplementary Material

Associations between EBV and CMV seropositivity, early exposures and gut microbiota in a prospective birth cohort: a 10 year follow-up

Claudia Carvalho-Queiroz^1^, Maria A. Johansson^1#^, Jan-Olov Persson^2#^, Evelina Jörtsö^3, 4^, Torbjörn Kjerstadius^5, 6^, Caroline Nilsson^3, 4^, Shanie Saghafian-Hedengren^7§^ and Eva Sverremark-Ekström^1§*^

*** Correspondence:** Eva Sverremark-Ekström, Stockholm University, Department of Molecular Bioscience, The Wenner-Gren Institute, Svante Arrhenius väg 20 C, 106 91 Stockholm, Sweden, Telephone: +46 8 16 41 78, Fax: +46 8 612 95 42, E-mail: eva.sverremark@ su.se

# Supplementary Table S2

| **Table S2: Relationship Between Children’s Early-Life Exposures and CMV Serostatus*** | | | | | | | | |  |
| --- | --- | --- | --- | --- | --- | --- | --- | --- | --- |
|  |  | 1Y |  |  |  | 2Y |  |  |  |
| Variable# | *N* | OR (95% CI) | *P* | *P_adj_* | *N* | OR (95% CI) | *P* | *P_adj_* |  |
| **Maternal age** | 115 | 1.00 (0.86, 1.16) | .99 | .47 | 253 | 0.94 (0.89, 1.00) | .04 | .35 |  |
| **Delivery mode** | 100 | 2.04 (0.38, 10.9) | .40 | .79 | 253 | 1.41 (0.71 2.80) | .33 | .20 |  |
| **Day-care start** | 94 | 0.80 (0.60, 1.07) | .14 | .10 | 220 | 0.98 (0.89, 1.07) | .59 | .81 |  |
| **Exclusive breastfeeding** | 115 | 0.97 (0.65, 1.46) | .89 | .50 | 253 | 0.99 (0.85, 1.16) | .95 | .83 |  |
| **Older Siblings** | 107 | 0.55 (0.17, 1.80) | .32 | .49 | 246 | 0.52 (0.35, 0.77) | **.001** | **.005** |  |
|  |  |  |  |  |  |  |  |  |  |
|  |  | 5Y |  |  |  | 10Y |  |  |  |
| Variable# | *N* | OR (95% CI) | *P* | *P_adj_* | *N* | OR (95% CI) | *P* | *P_adj_* |  |
| **Maternal age** | 224 | 0.94 (0.88, 1.00) | .04 | .03 | 207 | 0.93 (0.87, 0.99) | .03 | .04 |  |
| **Delivery mode** | 224 | 0.97 (0.47, 2.00) | .94 | .74 | 207 | 1.87 (0.80, 4.35) | .15 | .05 |  |
| **Day-care start** | 194 | 1.05 (0.95, 1.15) | .33 | .25 | 181 | 1.01 (0.91, 1.11) | .90 | .97 |  |
| **Exclusive breastfeeding** | 224 | 0.96 (0.81, 1.14) | .66 | .19 | 207 | 1.12 (0.94, 1.33) | .20 | .28 |  |
| **Older Siblings** | 210 | 0.66 (0.46, 0.94) | .02 | .08 | 194 | 0.67 (0.47, 0.97) | .03 | .10 |  |
| *Univariate analysis of association, for each age separately. Y: Years of age; N: Number of observations; OR: Odds ratio;  CI: Confidence interval range; *P_adj_*: values adjusted for included variables^#^. Bolded *P*-values: statistically significant if *P*<0.01 following Bonferroni correction. | | | | | | | | |  |
