# Supplementary material for: Associations between EBV and CMV Seropositivity, Early Exposures, and Gut Microbiota in a Prospective Birth Cohort: A 10-Year Follow-up
Source: Front Pediatr. 2016 Aug 31;4:93. doi: 10.3389/fped.2016.00093 (PMC5006634; doi:10.3389/fped.2016.00093)
Supplement: Supplementary file 3 [file Table_3.DOCX]

Supplementary Material

Associations between EBV and CMV seropositivity, early exposures and gut microbiota in a prospective birth cohort: a 10 year follow-up

Claudia Carvalho-Queiroz^1^, Maria A. Johansson^1#^, Jan-Olov Persson^2#^, Evelina Jörtsö^3, 4^, Torbjörn Kjerstadius^5, 6^, Caroline Nilsson^3, 4^, Shanie Saghafian-Hedengren^7§^ and Eva Sverremark-Ekström^1§*^

*** Correspondence:** Eva Sverremark-Ekström, Stockholm University, Department of Molecular Bioscience, The Wenner-Gren Institute, Svante Arrhenius väg 20 C, 106 91 Stockholm, Sweden, Telephone: +46 8 16 41 78, Fax: +46 8 612 95 42, E-mail: eva.sverremark@ su.se

# Supplementary Table S3

| **Table S3: EBV Serostatus in Relation to Gut Microbiota Colonization*** | | | | | | | | | | | | | | | | | | |
| --- | --- | --- | --- | --- | --- | --- | --- | --- | --- | --- | --- | --- | --- | --- | --- | --- | --- | --- |
| Detection |  | 1Y |  |  |  | 2Y |  |  |  | 5Y |  |  |  | 10Y |  |  | | |
| after birth | N | OR (95% CI) | *P* | *P_adj_* | N | OR (95% CI) | *P* | *P_adj_* | N | OR (95% CI) | *P* | *P_adj_* | N | OR (95% CI) | *P* | *P_adj_* | | |
| **Lactobacilli** |  |  |  |  |  |  |  |  |  |  |  |  |  |  |  |  | | |
| 1 week | 43 | 0.38 (0.07, 2.10) | .27 | .22 | 59 | 0.33 (0.07, 1.66) | .18 | .24 | 57 | 0.69 (0.21, 2.27) | .55 | .64 | 57 | 0.96 (0.27, 3.42) | .95 | .65 | | |
| 2 weeks | 42 | 0.25 (0.03, 2.26) | .22 | .18 | 58 | 0.84 (0.22, 3.13) | .79 | .85 | 54 | 0.61 (0.17, 2.16) | .45 | .42 | 55 | 2.44 (0.71, 8.46) | .16 | .08 | | |
| 1 month | 40 | 0.27 (0.03, 2.51) | .25 | .22 | 54 | 1.92 (0.50, 7.44) | .34 | .34 | 52 | 0.93 (0.29, 3.02) | .91 | .86 | 52 | 1.90 (0.59, 6.17) | .28 | .20 | | |
| 2 months | 41 | 0.40 (0.07, 2.28) | .30 | .30 | 57 | 0.86 (0.26, 2.91) | .81 | .64 | 54 | 0.60 (0.20, 1.80) | .36 | .22 | 54 | 0.72 (0.24, 2.13) | .55 | .45 | | |
| Occasions, ≥2 | 43 | 0.19 (0.02, 1.72) | .14 | .09 | 59 | 0.73 (0.19, 2.75) | .64 | .80 | 56 | 0.42 (0.13, 1.35) | .15 | .24 | 56 | 1.06 (0.35, 3.21) | .92 | .61 | | |
|  |  |  |  |  |  |  |  |  |  |  |  |  |  |  |  |  | | |
| ***S. aureus*** |  |  |  |  |  |  |  |  |  |  |  |  |  |  |  |  | | |
| 1 week | 43 | 0.97 (0.23, 4.14) | .97 | .99 | 59 | 0.59 (0.18, 1.94) | .39 | .33 | 57 | 1.17 (0.40, 3.37) | .78 | .98 | 57 | 0.67 (0.23, 1.98) | .47 | .29 | | |
| 2 weeks | 42 | 1.00 (0.21, 4.78) | 1 | .97 | 58 | 1.33 (0.36, 4.92) | .67 | .70 | 54 | 2.91 (0.86, 9.86) | .09 | .11 | 55 | 2.52 (0.77, 8.24) | .13 | .19 | | |
| 1 month | 40 | 0.84 (0.14, 5.11) | .85 | .91 | 54 | 1.36 (0.25, 7.37) | .72 | .76 | 52 | 2.25 (0.60, 8.45) | .23 | .34 | 52 | 1.40 (0.38, 5.16) | .61 | .92 | | |
| 2 months | 41 | 0.72 (0.14, 3.63) | .69 | .71 | 57 | 1.59 (0.38, 6.66) | .52 | .53 | 54 | 3.43 (0.94, 12.5) | .06 | .09 | 54 | 1.29 (0.40, 4.16) | .67 | .97 | | |
| Occasions, ≥2 | 46 | 0.58 (0.13, 2.49) | .46 | .46 | 62 | 1.05 (0.28, 3.90) | .94 | .93 | 59 | 2.48 (0.75, 8.22) | .14 | .24 | 60 | 1.18 (0.38, 3.63) | .77 | .81 | | |
| *Univariate analysis of associations, for each age group separately. N: Number of observations; OR: Odds ratio; CI: Confidence interval range. *P_adj_*: values adjusted for maternal age. Bolded *P*-values: statistically significant if *P*≤0.05. All subjects were vaginally delivered, fully breastfed for a minimum of 3 months and did not undergo any antibiotics treatment at the time of faecal sampling. | | | | | | | | | | | | | | | | |  |  |
