# Supplementary material for: Associations between EBV and CMV Seropositivity, Early Exposures, and Gut Microbiota in a Prospective Birth Cohort: A 10-Year Follow-up
Source: Front Pediatr. 2016 Aug 31;4:93. doi: 10.3389/fped.2016.00093 (PMC5006634; doi:10.3389/fped.2016.00093)
Supplement: Supplementary file 4 [file Table_4.DOCX]

Supplementary Material

Associations between EBV and CMV seropositivity, early exposures and gut microbiota in a prospective birth cohort: a 10 year follow-up

Claudia Carvalho-Queiroz^1^, Maria A. Johansson^1#^, Jan-Olov Persson^2#^, Evelina Jörtsö^3, 4^, Torbjörn Kjerstadius^5, 6^, Caroline Nilsson^3, 4^, Shanie Saghafian-Hedengren^7§^ and Eva Sverremark-Ekström^1§*^

*** Correspondence:** Eva Sverremark-Ekström, Stockholm University, Department of Molecular Bioscience, The Wenner-Gren Institute, Svante Arrhenius väg 20 C, 106 91 Stockholm, Sweden, Telephone: +46 8 16 41 78, Fax: +46 8 612 95 42, E-mail: eva.sverremark@ su.se

# Supplementary Tables S4

| **Table S4: CMV Serostatus in Relation to Gut Microbiota Colonization*** | | | | | | | | | | | | | | | | | | |
| --- | --- | --- | --- | --- | --- | --- | --- | --- | --- | --- | --- | --- | --- | --- | --- | --- | --- | --- |
| Detection |  | 1Y |  |  |  | 2Y |  |  |  | 5Y |  |  |  | 10Y |  |  | | |
| after birth | N | OR (95% CI) | *P* | *P_adj_* | N | OR (95% CI) | *P* | *P_adj_* | N | OR (95% CI) | *P* | *P_adj_* | N | OR (95% CI) | *P* | *P_adj_* | | |
| **Lactobacilli** |  |  |  |  |  |  |  |  |  |  |  |  |  |  |  |  | | |
| 1 week | 28 | 1 (1, 1) | 1 | 1 | 65 | 0.51 (0.14, 1.81) | .30 | .20 | 58 | 0.35 (0.11, 1.13) | .08 | .09 | 57 | 0.27 (0.06, 1.12) | .07 | .08 | | |
| 2 weeks | 42 | 1 (1, 1) | 1 | 1 | 63 | 0.63 (0.19, 2.06) | .44 | .35 | 56 | 0.52 (0.16, 1.68) | .27 | .29 | 55 | 0.72 (0.22, 2.35) | .58 | .57 | | |
| 1 month | 40 | 1 (1, 1) | 1 | 1 | 60 | 0.99 (0.86, 1.14) | .70 | .77 | 53 | 0.89 (0.77, 1.03) | .15 | .15 | 52 | 0.91 (0.77, 1.07) | .38 | .38 | | |
| 2 months | 41 | 1.47 (0.19, 11.6) | .71 | .72 | 62 | 1.21 (0.42, 3.52) | .73 | .70 | 56 | 0.57 (0.20, 1.65) | .30 | .27 | 54 | 0.72 (0.24, 2.13) | .55 | .54 | | |
| Occasions, ≥2 | 43 | 1 (1, 1) | 1 | 1 | 64 | 1.17 (0.39, 3.45) | .78 | .95 | 58 | 0.49 (0.17, 1.45) | .20 | .25 | 56 | 0.50 (0.16, 1.54) | .23 | .23 | | |
|  |  |  |  |  |  |  |  |  |  |  |  |  |  |  |  |  | | |
| ***S. aureus*** |  |  |  |  |  |  |  |  |  |  |  |  |  |  |  |  | | |
| 1 week | 43 | 2.09 (0.2, 21.91) | .54 | .58 | 65 | 1.54 (0.52, 4.55) | .43 | .36 | 58 | 1.69 (0.59, 4.84) | .33 | .37 | 57 | 2.03 (0.68, 6.10) | .21 | .22 | | |
| 2 weeks | 42 | 1 (1, 1) | 1 | 1 | 63 | 1.78 (0.54, 5.82) | .34 | .29 | 56 | 3.16 (1.00, 10.0) | **.05** | **.05** | 55 | 4.11 (1.2, 14.13) | **.02** | **.02** | | |
| 1 month | 40 | 1 (1, 1) | 1 | 1 | 60 | 1.37 (0.37, 5.05) | .63 | .51 | 53 | 2.06 (0.61, 6.97) | .24 | .28 | 52 | 2.44 (0.63, 9.45) | .20 | .18 | | |
| 2 months | 41 | 1.44 (0.14, 15.3) | .76 | .76 | 62 | 1.50 (0.45, 4.97) | .51 | .41 | 56 | 1.46 (0.48, 4.43) | .51 | .57 | 54 | 1.29 (0.40, 4.16) | .67 | .71 | | |
| Occasions, ≥2 | 46 | 1 (1, 1) | 1 | 1 | 68 | 1.65 (0.51, 5.31) | .41 | .32 | 61 | 2.12 (0.71, 6.29) | .18 | .21 | 60 | 2.32 (0.72, 7.41) | .16 | .16 | | |
| *Univariate analysis of associations, for each age group separately. N: Number of observations; OR: Odds ratio; CI: Confidence interval range. : values adjusted for maternal age. Bolded *P*-values: statistically significant if *P*≤0.05. All subjects were vaginally delivered, fully breastfed for a minimum of 3 months and did not undergo any antibiotics treatment at the time of faecal sampling. | | | | | | | | | | | | | | | | |  |  |
