# Supplementary material for: Associations between EBV and CMV Seropositivity, Early Exposures, and Gut Microbiota in a Prospective Birth Cohort: A 10-Year Follow-up
Source: Front Pediatr. 2016 Aug 31;4:93. doi: 10.3389/fped.2016.00093 (PMC5006634; doi:10.3389/fped.2016.00093)
Supplement: Supplementary file 5 [file Image_1.PDF]

Supplementary Data

Supplementary Figure 1 - Socioeconomic status of the children who become herpesvirus infected early versus those who are seronegative at age 10 years.

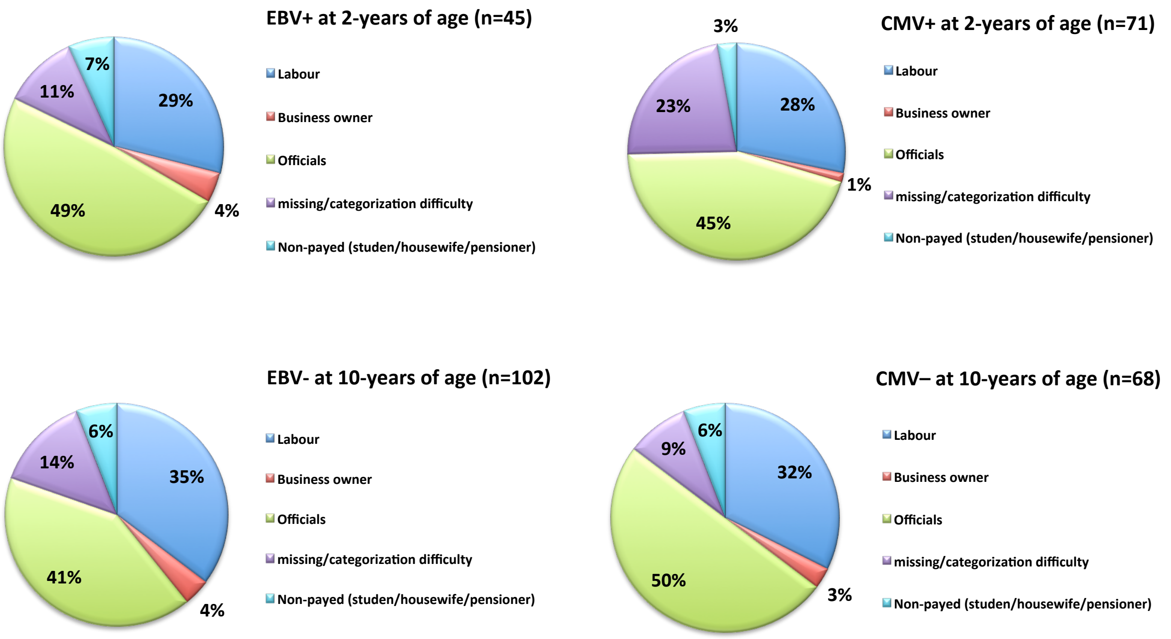

Socioeconomic index (SEI) of children's households according to categorization recommended by Statistics Sweden (http://www.scb.se/en). Demographic data was available from n=147 (for EBV) and n=139 (for CMV) subjects on the basis of early (seropositive at age of 2 years) or absence of infection (seronegative at age of 10 years).
